# Single-Incision Laparoscopic Liver Resection for Colorectal Metastasis through Stoma Site at Time of Reversal of Diversion Ileostomy: A Case Report

**DOI:** 10.1155/2011/502176

**Published:** 2011-08-01

**Authors:** Bård I. Røsok, Bjørn Edwin

**Affiliations:** ^1^Section of Gastrointestinal and Hepatobiliary Surgery, Clinic for Specialized Medicine and Surgery, Oslo University Hospital-Rikshospitalet, Sognsvannsveien 20, P.O. Box 4950 Nydalen, 0424 Oslo, Norway; ^2^The Interventional Center, Clinic for Visualization and Interventional Medicine, Oslo University Hospital-Rikshospitalet, P.O. Box 4950 Nydalen, 0424 Oslo, Norway

## Abstract

Minimally invasive surgical techniques for liver tumors are gaining increased acceptance as an alternative to traditional resections by laparotomy. In this article we describe a laparoscopic liver resection of a metastatic lesion in a patient primarily operated for colorectal cancer. The resection was conducted as a single port procedure through the stoma aperture at time of reversal of the diversion ileostomy. Sigle incision liver resections may be less traumatic than conventional laparoscopy and could be applied in selected patients with both benign and malignant liver tumors.

## 1. Introduction

Laparoscopic liver resection has gained increased acceptance as an alternative to liver resections by laparotomy, even for malignant tumors [1]. In our department, liver resections have been done by the laparoscopic approach for more than a decade, and our series for both benign and malignant liver tumors comprises one of the largest single centre experiences published [1, 2]. In this paper, we describe a patient with a single, synchronous liver metastasis from a colorectal adenocarcinoma operated by a single-port laparoscopic access through the stoma site of at time of reversal of the diversion loop ileostomy.

## 2. Patient and Methods

**Figure 1:**
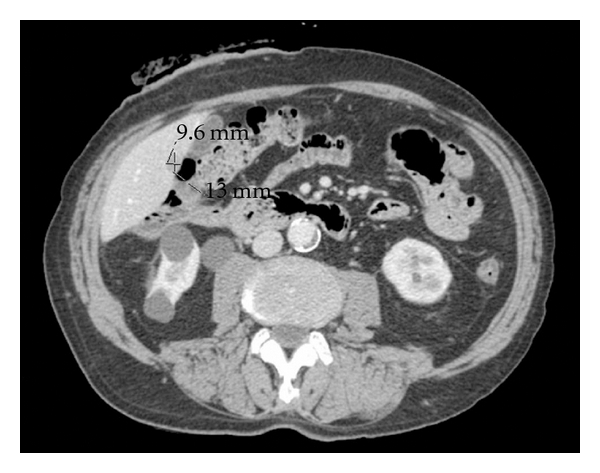
CT scan showing the metastasis located in segment 5, in close relation to the gall bladder.

The patient was a 79-year-old man with concomitant pre-dialytic kidney failure who was initially operated for two synchronous adenocarcinomas of which one was located in the ascending colon and the other at the rectosigmoid junction. The primary operation was done by the laparoscopic approach with a right-sided hemicolectomy and a separate low anterior total mesorectal resection of the rectum. A diversion loop ileostomy was constructed. There were no locoregional lymph node metastases detected in any of the resected primary tumor specimens, and the patient did not receive adjuvant chemotherapy, in accordance with Norwegian national guidelines for colorectal cancer [3]. A small, synchronous liver metastasis was detected at time of colorectal surgery, classifying the patient with stage IV disease. The 15 mm tumor was located subcapsular in segment 5 on the posterior aspect of the liver immediately lateral to the gallbladder (Figure 1). The patient was referred to our hospital for liver resection 6 months after surgery for the primary tumors. The delay was due to a prolonged postoperative course following the colorectal resections.

The liver resection was planned as a combined procedure in combination with reversal of the loop ileostomy. 

**Figure 2:**
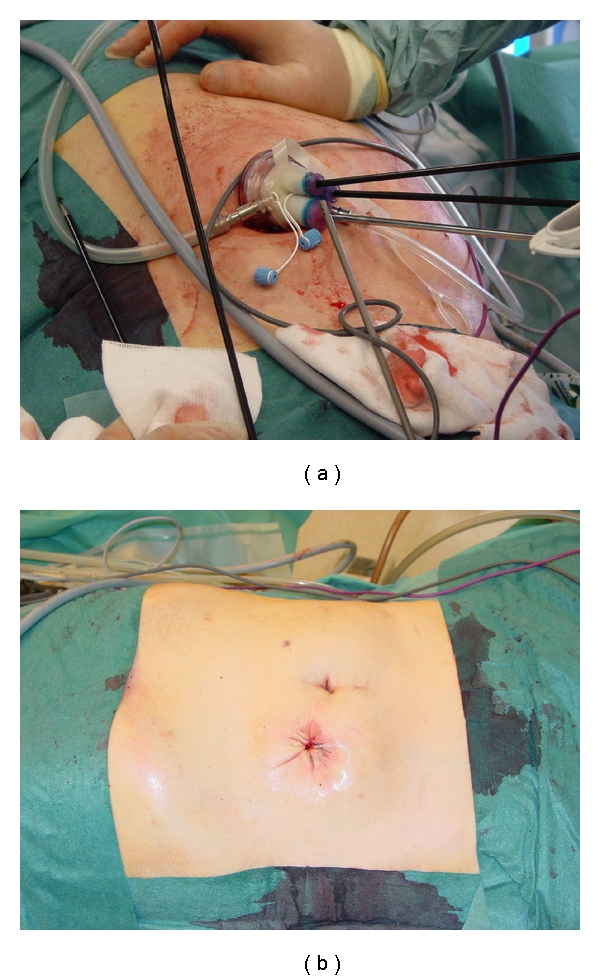
(a) Instrumentation through the LESS port. (b) Postoperative cosmetic result showing a single scar.

The patient was placed in a prone position. The ileostomy was dissected free from the surrounding tissue. A small bowel resection was necessary, and an end-to-end anastomosis was made. After completion of the anastomosis, a Laparo-Endoscopic Single-Site (LESS) tri-port trocar (Olympus) was introduced through the stoma site. Pneumoperitoneum was established at 10 mmHg. A percutaneous suture was introduced in the epigastrium and secured in the fissure between segments 3 and 4 in order to retract the liver upwards for proper visualisation of the tumor. A 5 mm Deflectable-Tip EndoEYE camera (Olympus) was used for visualization as were specially designed curved instruments to obtain adequate exposure and triangulation. Instrumentation is shown in Figure 2(a). The resection margins were determined by intraoperative ultrasonography (Aloca, Wallingford, CT), the liver capsula was divided by an ultrasonic cutting and coagulation device (SonoSurg, Olympus), and the liver parenchyma was divided by the LigaSure (Covidien) bipolar tissue sealing device as previously described [2]. Intraoperative bleeding was 120 mL. Tumor margins were free with a minimum distance of 5 mm. The stoma aperture was closed with two separate layers of fascia suture and the skin was closed by a continous pursestring suture (Figure 2(b)).

No surgical complications occurred but due to a misfortunate postoperative fluid overload in combination with the patients pre-existing kidney failure, he developed a moderate pulmonary oedema which was resolved in two days with temporary respiratory support. He was discharged to his local hospital on the 5th postoperative day and went home seven days after surgery in good condition.

The patient has been observed with repeated CT scans for 6 months without any evidence of tumor recurrence.

## 3. Discussion

Laparoscopic liver resection has proven safe and feasible and can be performed according to established oncological principles in institutions with experience in both hepatobiliary and advanced laparoscopic surgery [1]. The minimally invasive approach has several documented advantages such as fast recovery and cosmetic superiority and may even have some immunological benefits in malignant disease. The development of modern surgical tools has enabled us to perform these resections with minimal bleeding and excellent visual control. Our recently published series of laparoscopic liver resection has shown that such resections can be performed with excellent perioperative results and oncological outcome compared to traditional, open surgery. The search for even less invasive methods the last few years has led to the development of Natural Orifice Transluminal Endoscopic Surgery (NOTES) techniques as well as the development of equipment enabling surgery through single incisions. Several companies now deliver specially designed products for such procedures. To date, a wide variety of single incision surgical procedures have been reported, including cholecystectomy, appendectomy, adrenalectomy, splenectomy, and colectomy. Liver resections by the single incision have been scarely reported and certainly not as a simultaneous procedure through a bowel stoma site following reversal of a loop enterostomy. 

It is obvious that not all metastatic lesions of the liver are suitable for this technique. In our experience, the preferred lesions would be superficially located on the anterior aspect of the liver. Such lesions will not demand extensive triangulation, major mobilization, or retraction of the remnant liver. The technique is also suitable for smaller anatomical resections such as resection of segment 2/3 as suggested in a recent publication [4]. 

The ideal timing from resection of a synchronous liver metastasis from a colorectal carcinoma is not known. Neo-adjuvant or adjuvant chemotherapy is gaining acceptance as standard of care in many institutions, and recently published data indicates increased long-term survival and longer disease-free survival following this approach [5, 6]. In the present case report, a neoadjuvant chemotherapy algorithm had not been implemented in our institution, but the patient's age and pre-existing renal failure would in any case have contraindicated chemotherapy. 

Although simultaneous surgery for a primary colorectal adenocarcinoma and combined liver surgery may be considered safe in selected cases, many centers still choose a two-stage procedure [7]. In patients with high risk of anastomosis complications, which may be the case in some low anterior resections for rectal cancer, one may consider performing a laparoscopic liver resection prior to resection of the primary tumor in order to prevent delay in the treatment of liver metastases. However, the optimal strategy for resectable synchronous metastases from colorectal cancer is still not well defined. If a two-stage procedure is selected and a loop ileostomy has been established during the primary surgery, the single-port access for liver resection could be of particular interest in selected patients in centers with experience in laparoscopic liver resection, to minimize the surgical trauma to the abdominal wall. The position of an ileostoma in the right lower quadrant provides excellent visualisation of the anterior aspect of segments 4b, 5, 8 and the lower lateral parts of segment 6, and the distance from the stoma site to these segments facilitates adequate working conditions with available single-port equipment.

In this case, the patient was fully mobilized on the day of his surgery and was scheduled for dismissal on the second postoperative day. Before discharge, however, he suffered a respiratory complication. His pre-existing kidney failure was most likely underestimated, and due to a relatively low urine output he was given excess crystalloids without proper concomitant administration of diuretics. After proper treatment for the subsequent, transient pulmonary edema, his recovery went uneventful. We believe that the respiratory complication was related to his underlying renal condition and not to the surgical technique. 

Further studies are needed in order to determine this method's potential position among other minimally invasive liver resection techniques.
